# Prognostic Significance of Admission Systemic Inflammation Response Index in Patients With Spontaneous Intracerebral Hemorrhage: A Propensity Score Matching Analysis

**DOI:** 10.3389/fneur.2021.718032

**Published:** 2021-09-24

**Authors:** Junhong Li, Yunbo Yuan, Xiang Liao, Zhiyuan Yu, Hao Li, Jun Zheng

**Affiliations:** ^1^Department of Neurosurgery, West China Hospital of Sichuan University, Chengdu, China; ^2^Department of Cardiology, PLA Rocket Force Characteristic Medical Center, Beijing, China

**Keywords:** systemic inflammation response index, neutrophil to lymphocyte ratio, intracerebral hemorrhage, prognosis, propensity score matching

## Abstract

Intracerebral hemorrhage (ICH) accounts for ~15% of all strokes and is associated with high mortality and disability rates. The systemic inflammation response index (SIRI) is a novel systemic inflammatory marker based on peripheral neutrophil, monocyte, and lymphocyte counts. This study aimed to evaluate the prognostic significance of admission SIRI in patients with spontaneous ICH and compare its predictive ability with that of the neutrophil-to-lymphocyte ratio (NLR). This retrospective study was conducted based on a prospectively collected database of patients with ICH between June 2016 and January 2019. Propensity score matching (PSM) was conducted to adjust for potential imbalances in the clinical parameters. A total of 403 patients were included in the original cohort. The optimal SIRI cut-off value was 2.76. After 1:1 PSM based on potential confounding variables, a new cohort containing 262 patients was established for further analysis. In the original cohort, SIRI served as an independent predictor of 3-month functional outcome [odds ratio (OR), 1.302; 95% CI, 1.120–1.512; *p* = 0.001] and 1-month mortality (OR, 1.072; 95% CI, 1.020–1.126; *p* = 0.006), while NLR was independently associated with only 3-month functional outcomes (OR, 1.051; 95% CI, 1.004–1.100; *p* = 0.031) and not 1-month mortality. The same applied to the PSM cohort. Receiver operating characteristic analyses and predictive models indicated that in most instances, SIRI was superior to NLR and their components in predicting the outcomes of patients with ICH. Our study found that SIRI is determined to be an independent predictive indicator for ICH patients in 3-month functional outcomes and 1-month mortality. The prognostic predictive ability of SIRI was stronger than that of NLR.

## Introduction

Intracerebral hemorrhage (ICH) is a life-threatening condition with a high mortality and disability rate and occurs due to spontaneous bleeding into the brain parenchyma, involving the ventricles and subarachnoid spaces in extreme circumstances. ICH accounts for ~15% of all strokes (1). In terms of ICH, 75–85% of cases originate from the spontaneous rupture of small vessels damaged. by chronic hypertension or amyloid angiopathy (2). The incidence of ICH is higher in male and elderly patients. Rapid CT after onset can be used to recognize almost all forms of acute ICH and help make optimal medical decisions within the shortest time. The global burden of ICH mainly results from inadequate management of chronic hypertension and other modifiable risk factors (3)

Growing evidence has indicated that inflammatory responses participate in the pathophysiological processes of brain injury after ICH, and inflammation is one of the crucial contributors to ICH-induced secondary brain injury (4). Leukocytes play an important role in immune response, cell migration, perihematomal edema formation, blood–brain barrier (BBB) integrity, and cell death after ICH (5, 6). Accumulating data have demonstrated that increased blood leukocyte count is associated with more serious disease and worse outcomes in ischemic and hemorrhagic strokes (7). Neutrophil-to-lymphocyte ratio (NLR), based on the coexistence of lymphopenia and leukocytosis in the initial inflammatory response, may be a useful peripheral biomarker for predicting the prognosis of stroke (8). Other peripheral inflammatory biomarkers, whose prognostic ability in ICH patients has also been confirmed, are systemic immune-inflammation index, NLR, and platelet-to-lymphocyte ratio (9, 10). Systemic inflammatory response syndrome, which is defined based on the changes in leukocyte and vital signs, is also associated with outcomes (11, 12).

The systemic inflammation response index (SIRI) is a novel systemic inflammatory marker based on peripheral neutrophil, monocyte, and lymphocyte counts. In previous studies, SIRI was found to be an independent prognostic indicator in various tumors (13–15). Therefore, this study aimed to evaluate the prognostic significance of admission SIRI in patients with spontaneous ICH and compare its prognostic ability with that of NLR.

## Materials and Methods

### Study Design

This retrospective study was conducted based on a prospectively collected database of ICH patients at the Department of Neurosurgery of West China Hospital, Sichuan University between June 2016 and January 2019. All patients in this cohort were managed according to the latest guidelines for stroke, and their baseline clinical data were retrieved from the electronic medical record system of the West China Hospital (16).

The exclusion criteria were as follows: (1) age <18 years; (2) incomplete baseline clinical data; (3) ICH caused by a tumor, aneurysm, or arteriovenous malformation; (4) absence of CT angiography and follow-up CT within 24 h of admission; (5) a history of infectious diseases, cancers, rheumatic diseases, blood system diseases, or other diseases which evidently affect peripheral blood cells; (6) loss to follow-up.

### Clinical Parameter Assessment

Clinical variables were retrieved from the electronic medical record system, including the following variables: (1) demographics: age of onset and sex; (2) clinical history: history of hypertension, diabetes mellitus, smoking, alcohol abuse, and stroke; (3) admission conditions: Glasgow Coma Scale (GCS) score, admission systolic blood pressure, diastolic blood pressure, and duration from onset to hospitalization; (4) ICH imaging characteristics: hematoma volume, location of hematoma, presence of intraventricular hematoma, and hematoma expansion (HE); (5) treatment; (6) routine blood tests. Notably, routine blood tests were conducted immediately after admission. SIRI was defined as neutrophil count × monocyte count/lymphocyte count, and NLR was defined as neutrophil count/lymphocyte count.

Patients were followed up every month after admission. The primary outcomes were 3-month functional outcomes and 1-month mortality rate. The modified Rankin Scale (mRS) was used to evaluate patients' functional outcomes at each follow-up. Patients who had been discharged were followed up by telephone. Good outcome was defined as an mRS score of 0–2, while a poor outcome was defined as an mRS score of 3–6 (17).

The volume of parenchymal hematoma was calculated on the initial CT scans using 3D Slicer (http://www.slicer.org), and manual segmentation was applied by two independent neurosurgeons (18). HE was defined as hematoma enlargement ≥6 ml or ≥33% within 24 h (19). Surgical interventions mainly included hematoma evacuation with craniotomy and external ventricular drainage.

### Statistical Analysis

All statistical analyses were performed using SPSS software (version 22.0; IBM, Armonk, NY, USA) and R software (version 3.6.1). Continuous variables are presented as mean ± SD or median with interquartile range, while categorical variables are presented as frequency and percentage. Categorical variables were compared using the χ^2^ or Fisher's exact test. Continuous variables that conformed to the normal distribution were compared using Student's *t*-test; otherwise, the Mann–Whitney *U*-test was employed. Logistic regression analyses were used to determine the influence of risk factors on outcomes in patients with ICH. Variables with *p* < 0.1 in univariate analysis were included in backward stepwise multivariate logistic regression. Receiver operating characteristic (ROC) analysis was conducted to assess the accuracy of the SIRI, NLR, and other markers for outcomes. The optimal cut-off value of SIRI was determined by calculating the maximum Youden index using ROC. DeLong's test was employed to compare the areas under the curve (AUC). Predictive models for outcomes were constituted by independent predictive indicators in multivariate logistic regression; Harrell's concordance index (C-index) and Akaike information criterion (AIC) were used to assess the predictive accuracy and model-fitting of predictive models, respectively. Higher C-index indicated better predictive accuracy, and lower AICs indicated superior model-fitting (20, 21). A two-sided *p* < 0.05 was considered statistically significant. Propensity score matching (PSM) was conducted to adjust for an imbalance of clinical parameters with a *p*-value of <0.1 in univariate analysis. These patients were matched 1:1 using the nearest-neighbor algorithm with a caliper width of 0.2 and without replacement.

### Ethics

This study was approved by the Ethical Committee of Sichuan University (2013NO52) and conducted following the principles of the Declaration of Helsinki. All patients and their authorized trustees were informed and provided signed informed consent to use their clinical data for research purposes.

## Results

### Baseline Clinical Characteristics

**Figure 1:**
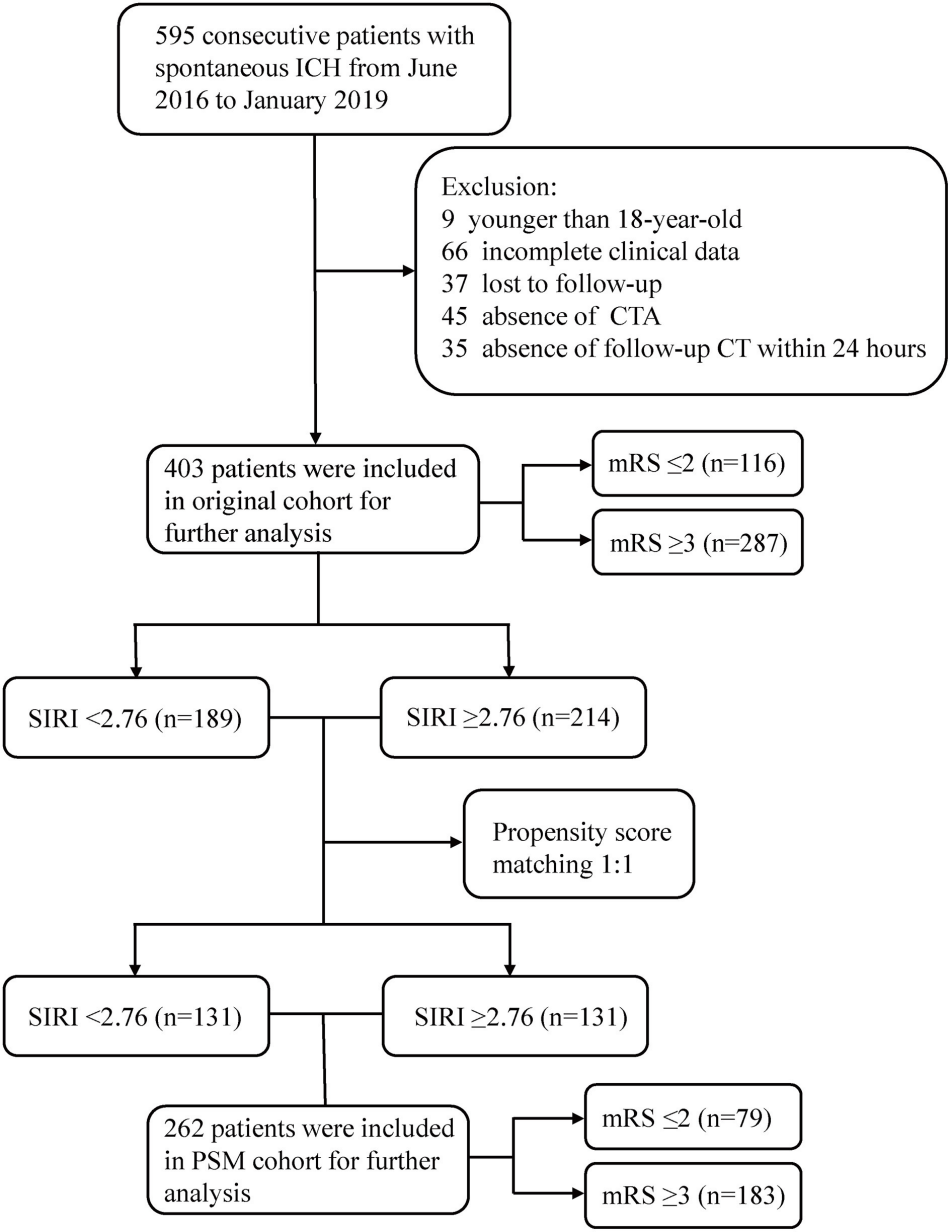
Flow chart of the current study. ICH, intracerebral hemorrhage; CTA, computed tomography angiography; mRS, modified Rankin Scale; SIRI, systemic inflammation response index; PSM, propensity score matching.

As shown in Figure 1, a total of 403 patients were included in the original cohort. The optimal cut-off value of SIRI was determined to be 2.76 in ROC analysis. Among the 403 patients, 189 patients had SIRI <2.76 and 214 had SIRI ≥2.76. After 1:1 PSM based on potential confounding variables, a new cohort containing 262 patients was established for further analysis.

In the original cohort (Table 1), 287 patients had poor outcomes at 3 months with mRS score ≥3, while 116 patients had good outcomes with mRS score ≤ 2. Clinical variables including GCS score (*p* < 0.001), hematoma volume (*p* < 0.001), hematoma location (*p* = 0.010), presence of IVH (*p* = 0.018), presence of HE (*p* = 0.003), and treatment method (*p* < 0.001) were associated with 3-month functional outcomes. Meanwhile, neutrophil count (*p* < 0.001), lymphocyte count (*p* = 0.004), monocyte count (*p* < 0.001), NLR (*p* < 0.001), and SIRI (*p* < 0.001) were also associated with 3-month functional outcomes. Regarding 1-month mortality, 84 patients died within 30 days of admission, and 319 patients survived the first month. Among the clinical variables, GCS score (*p* < 0.001), duration from onset to hospitalization (*p* = 0.006), hematoma volume (*p* = 0.002), and presence of HE (*p* = 0.004) were associated with 1-month mortality. Peripheral blood markers, including neutrophil count (*p* < 0.001), monocyte count (*p* < 0.001), NLR (*p* < 0.001), and SIRI (*p* < 0.001), were significantly associated with 1-month mortality, whereas lymphocyte count (*p* = 0.131) was not associated with 1-month mortality.

**Table 1:** Baseline characteristics of 403 patients with spontaneous ICH in original cohort.

**Clinical variables**	**Total (*n =* 403)**	**3-month functional outcome**			**1-month mortality**		
		**Poor outcome**	**Good outcome**	***P*-value**	**Death**	**Survival**	***P*-value**
		**(*n =* 287)**	**(*n =* 116)**		**(*n =* 84)**	**(*n =* 319)**	
Age (years)	58.56 ± 13.28	59.35 ± 13.31	56.60 ± 13.07	0.172	60.64 ± 13.90	58.01 ± 13.08	0.385
Male	276 (68.5)	196 (68.3)	80 (69.0)	0.895	56 (66.7)	220 (69.0)	0.687
GCS	13 (8, 15)	11 (7, 14)	15 (14, 15)	***<0.001***	7 (6, 9)	14 (11, 15)	***<0.001***
Admission SBP (mmHg)	167.34 ± 27.54	168.74 ± 28.55	163.87 ± 24.63	0.175	169.35 ± 30.84	166.81 ± 26.63	0.265
Admission DBP (mmHg)	98.10 ± 18.27	97.90 ± 19.11	98.61 ± 16.05	0.157	96.65 ± 20.12	98.49 ± 17.76	0.663
Duration from onset to hospitalization (h)	6 (4, 12)	6 (4, 10)	6 (4, 16)	0.395	5 (4, 7)	6 (4, 14)	***0.006***
History of hypertension	226 (56.1)	164 (57.1)	62 (53.4)	0.499	53 (63.1)	173 (54.2)	0.146
History of diabetes mellitus	25 (6.2)	21 (7.3)	4 (3.4)	0.145	3 (3.6)	22 (6.9)	0.262
Smoking	133 (33.0)	102 (35.5)	31 (26.7)	**0.089**	23 (27.4)	110 (34.5)	0.219
Alcohol abuse	127 (31.5)	95 (33.1)	32 (27.6)	0.281	21 (25.0)	106 (33.2)	0.149
Previous stroke	22 (5.5)	17 (5.9)	5 (4.3)	0.519	3 (3.6)	19 (6.0)	0.393
Antiplatelet or anticoagulation	30 (7.4)	18 (6.3)	12 (10.3)	0.159	5 (6.0)	25 (7.8)	0.293
Hematoma volume (ml)	18.36 (7.35, 36.33)	24.68 (9.18, 41.53)	10.19 (3.17, 20.51)	***<0.001***	25.99 (9.21, 45.36)	12.63 (7.05, 33.10)	***0.002***
Hematoma location							
Supratentorial	336 (83.4)	248 (86.4)	88 (75.9)	***0.010***	65 (77.4)	271 (85.0)	**0.098**
Infratentorial	67 (16.6)	39 (13.6)	28 (24.1)		19 (22.6)	48 (15.0)	
Presence of IVH	53 (13.2)	45 (15.7)	8 (6.9)	***0.018***	14 (16.7)	39 (12.2)	0.285
Presence of hematoma expansion	92 (22.8)	77 (26.8)	15 (12.9)	***0.003***	29 (34.5)	63 (19.7)	***0.004***
Treatment							
Surgical intervention	101 (25.1)	92 (32.1)	9 (7.8)	***<0.001***	24 (28.6)	77 (24.1)	0.405
Conservative treatment	302 (74.9)	195 (67.9)	107 (92.2)		60 (71.4)	242 (75.9)	
PLT (10^9^/L)	153 (117, 203)	154 (114, 204)	154 (132, 202)	0.592	144 (112, 197)	158 (122, 206)	0.201
PT (s)	11.4 (10.9, 12.2)	11.4 (10.8, 12.2)	11.4 (10.9, 12.1)	0.396	11.3 (10.9, 12.4)	11.4 (10.8, 12.1)	0.463
APTT (s)	26.5 (23.7, 29.4)	26.1 (23.4, 28.5)	26.7 (24.4, 29.7)	0.701	25.2 (22.1, 28.5)	26.3 (24.0, 29.0)	0.240
INR	0.98 (0.93–1.05)	0.98 (0.93, 1.06)	0.99 (0.93, 1.04)	0.800	0.98 (0.93, 1.10)	0.98 (0.92, 1.05)	0.223
Neutrophil (10^9^/L)	8.66 (6.19, 11.73)	9.68 (7.03, 12.98)	6.61 (4.88, 8.79)	***<0.001***	11.76 (8.66, 15.29)	8.00 (5.89, 10.52)	***<0.001***
Lymphocyte (10^9^/L)	1.03 (0.72, 1.48)	0.98 (0.69, 1.41)	1.18 (0.82, 1.62)	***0.004***	0.93 (0.64, 1.37)	1.05 (0.76, 1.49)	0.131
Monocyte (10^9^/L)	0.39 (0.27, 0.53)	0.42 (0.29, 0.56)	0.34 (0.23, 0.42)	***<0.001***	0.51 (0.41, 0.64)	0.36 (0.24, 0.49)	***<0.001***
NLR	8.63 (5.11, 13.96)	9.83 (6.23, 15.77)	5.71 (3.43, 9.58)	***<0.001***	12.16 (6.39, 21.04)	7.96 (4.66, 12.62)	***<0.001***
SIRI	2.87 (1.63, 5.56)	3.75 (2.10, 6.58)	1.87 (1.05, 2.89)	***<0.001***	5.68 (3.50, 10.13)	2.55 (1.50, 4.44)	***<0.001***

*Data are expressed as n (%), mean ± SD, or median (25th, 75th quartile). Significant findings are expressed in bold and italic*.

*ICH, intracerebral hemorrhage; GCS, Glasgow Coma Scale; SBP, systolic blood pressure; DBP, diastolic blood pressure; IVH, intraventricular hematoma; PLT, platelet; PT, prothrombin time; APTT, activated partial thromboplastin time; INR, international normalized ratio; SIRI, systemic inflammation response index; NLR, neutrophil-to-lymphocyte ratio*.

The clinical characteristics of the PSM cohort are listed in Table 2, with a lower GCS score (*p* < 0.001), larger hematoma volume (*p* < 0.001), presence of IVH (*p* = 0.037), supratentorial hematoma (*p* = 0.001), and surgical interventions (*p* = 0.001) associated with unfavorable outcomes at 3 months after admission. In the group with 1-month mortality, a lower GCS score (*p* < 0.001) and shorter duration from onset to hospitalization (*p* = 0.015) were directly related to death. Higher neutrophil count, monocyte count, and SIRI were associated with unfavorable outcomes in both groups. Higher NLR was significantly related to poor 3-month functional outcomes (*p* < 0.001) but not 1-month mortality (*p* = 0.271).

**Table 2:** Baseline characteristics of 262 patients with spontaneous ICH in PSM cohort.

**Clinical variables**	**Total (*n =* 262)**	**3-month functional outcome**			**1-month mortality**		
		**Poor outcome**	**Good outcome**	***P*-value**	**Death**	**Survival**	***P*-value**
		**(*n =* 183)**	**(*n =* 79)**		**(*n =* 42)**	**(*n =* 220)**	
Age (years)	58.77 ± 13.56	59.67 ± 13.42	56.67 ± 13.75	0.402	61.26 ± 14.82	58.29 ± 13.29	0.242
Male	177 (67.6)	124 (67.8)	53 (67.1)	0.915	28 (66.7)	149 (67.7)	0.893
GCS	13 (9, 15)	12 (8, 14)	15 (14, 15)	***<0.001***	8 (6, 11)	14 (11, 15)	***<0.001***
Admission SBP (mmHg)	167.17 ± 26.56	168.86 ± 27.25	163.24 ± 24.60	0.407	170.31 ± 27.05	166.57 ± 26.48	0.857
Admission DBP (mmHg)	97.56 ± 17.54	97.58 ± 17.86	97.49 ± 16.90	0.655	96.05 ± 18.81	97.85 ± 17.32	0.844
Duration from onset to hospitalization (h)	6 (4, 15)	6 (4, 12)	6 (4, 22)	0.242	5 (3, 8)	6 (4, 15)	***0.015***
History of hypertension	141 (53.8)	103 (56.3)	38 (48.1)	0.224	26 (61.9)	115 (52.3)	0.252
History of diabetes mellitus	14 (5.3)	13 (7.1)	1 (1.3)	0.054	1 (2.4)	13 (5.9)	0.352
Smoking	90 (34.4)	66 (36.1)	24 (30.4)	0.375	14 (33.3)	76 (34.5)	0.880
Alcohol abuse	88 (33.6)	67 (36.6)	21 (26.6)	0.115	13 (31.0)	75 (34.1)	0.694
Previous stroke	14 (5.3)	13 (7.1)	1 (1.3)	0.054	2 (4.8)	12 (5.5)	0.855
Antiplatelet or anticoagulation	21 (8.0)	13 (7.1)	8 (10.1)	0.409	3 (7.1)	18 (8.2)	0.821
Hematoma volume (ml)	20.10 (7.04, 35.15)	24.18 (9.61, 38.54)	10.13 (2.71, 24.02)	***<0.001***	24.87 (8.88, 39.99)	18.29 (7.00, 33.09)	0.136
Hematoma location							
Supratentorial	215 (82.1)	160 (87.4)	55 (69.6)	***0.001***	34 (81.0)	181 (82.3)	0.838
Infratentorial	47 (17.9)	23 (12.6)	24 (30.4)		8 (19.0)	39 (17.7)	
Presence of IVH	38 (14.5)	32 (17.5)	6 (7.6)	***0.037***	9 (21.4)	29 (13.2)	0.165
Presence of hematoma expansion	61 (23.3)	47 (25.7)	14 (17.7)	0.163	14 (33.3)	47 (21.4)	0.093
Treatment							
Surgical intervention	61 (23.3)	53 (29.0)	8 (10.1)	***0.001***	10 (23.8)	51 (23.2)	0.930
Conservative treatment	201 (76.7)	130 (71.0)	71 (89.9)		32 (76.2)	169 (76.8)	
PLT (10^9^/L)	148 (117, 196)	147 (116, 195)	153 (123, 187)	0.525	136 (112, 196)	153 (120, 196)	0.171
PT (s)	11.4 (10.9, 12.2)	11.4 (10.8, 12.3)	11.5 (11.0, 12.2)	0.651	11.6 (10.9, 12.3)	11.4 (10.9, 12.2)	0.703
APTT (s)	26.2 (23.5, 29.2)	25.9 (23.4, 28.9)	26.5 (23.9, 29.3)	0.650	24.8 (22.5, 29.8)	26.2 (23.6, 29.1)	0.511
INR	0.98 (0.93, 1.05)	0.98 (0.92, 1.05)	1.00 (0.96, 1.05)	0.169	0.99 (0.94, 1.10)	0.98 (0.93, 1.05)	0.421
Neutrophil (10^9^/L)	8.36 (6.37, 10.81)	9.06 (6.80, 11.35)	7.50 (5.49, 9.08)	***<0.001***	10.22 (7.19, 12.54)	8.19 (6.32, 10.39)	***0.017***
Lymphocyte (10^9^/L)	1.05 (0.75, 1.49)	1.04 (0.71, 1.44)	1.13 (0.82, 1.57)	0.085	1.04 (0.70, 1.60)	1.06 (0.76, 1.48)	0.965
Monocyte (10^9^/L)	0.38 (0.25, 0.49)	0.40 (0.28, 0.50)	0.34 (0.24, 0.46)	***0.044***	0.48 (0.40, 0.58)	0.36 (0.24, 0.47)	***<0.001***
NLR	8.18 (4.88, 12.67)	8.94 (5.91, 13.26)	6.41 (4.05, 9.95)	***<0.001***	10.31 (5.78, 14.86)	7.90 (4.87, 12.44)	0.271
SIRI	2.76 (1.63, 4.70)	2.99 (1.81, 5.20)	2.18 (1.30, 3.23)	***<0.001***	4.60 (1.95, 8.47)	2.62 (1.63, 4.15)	***0.003***

*Data are expressed as n (%), mean ± SD, or median (25th, 75th quartile)*.

*ICH, intracerebral hemorrhage; PSM, propensity score matching; GCS, Glasgow Coma Scale; SBP, systolic blood pressure; DBP, diastolic blood pressure; IVH, intraventricular hematoma; PLT, platelet; PT, prothrombin time; APTT, activated partial thromboplastin time; INR, international normalized ratio; SIRI, systemic inflammation response index; NLR, neutrophil-to-lymphocyte ratio. Significant findings are expressed in bold and italic*.

### Association of SIRI With Outcomes

In the original cohort, multivariate logistic analysis (Table 3) revealed that the following factors all served as independent predictors for 3-month functional outcomes, including GCS score [odds ratio (OR), 0.686; 95% CI 0.606–0.776; *p* < 0.001), hematoma volume (OR, 1.022; 95% CI 1.002–1.042; *p* = 0.027), hematoma location (OR, 2.452; 95% CI 1.100–5.467; *p* = 0.028), treatment method (OR, 2.455; 95% CI 1.044–5.773; *p* = 0.040), lymphocyte count (OR, 0.469; 95% CI 0.290–0.758; *p* = 0.002), monocyte count (OR, 28.642; 95% CI 4.427–185.296; *p* < 0.001), NLR (OR, 1.051; 95% CI 1.004–1.100; *p* = 0.031), and SIRI (OR, 1.302; 95% CI 1.120–1.512; *p* = 0.001). As for 1-month mortality, GCS score (OR, 0.700; 95% CI 0.637–0.769; *p* < 0.001), hematoma volume (OR, 1.012; 95% CI 1.000–1.025; *p* = 0.046), monocyte count (OR, 3.734; 95% CI 1.283–10.869; *p* = 0.016), and SIRI (OR, 1.072; 95% CI 1.020–1.126; *p* = 0.006) were independent risk factors, but not NLR (OR, 1.021; 95% CI 0.987–1.057; *p* = 0.225).

**Table 3:** Multivariate logistic regression of included clinical variables for 3-month functional outcome and 1-month mortality in original cohort.

**3-month functional outcome**	**OR**	**95% CI**	***P*-value**
GCS (per 1-point increase)	0.686	0.606–0.776	***<0.001***
Smoking (yes vs. no)	1.426	0.791–2.572	0.238
Hematoma volume (per 1-ml increase)	1.022	1.002–1.042	***0.027***
Hematoma location (supratentorial vs. infratentorial)	2.452	1.100–5.467	***0.028***
Presence of IVH (yes vs. no)	1.272	0.496–3.263	0.616
Presence of hematoma expansion (yes vs. no)	1.093	0.508–2.351	0.820
Treatment (surgical intervention vs. conservative treatment)	2.455	1.044–5.773	***0.040***
Neutrophil (per 1 × 10^9^/L increase)	1.080	0.981–1.189	0.118
Lymphocyte (per 1 × 10^9^/L increase)	0.469	0.290–0.758	***0.002***
Monocyte (per 1 × 10^9^/L increase)	28.642	4.427–185.296	***<0.001***
NLR (per 1-point increase)	1.051	1.004–1.100	***0.031***
SIRI (per 1-point increase)	1.302	1.120–1.512	***0.001***
**1-month mortality**			
GCS (per 1-point increase)	0.700	0.637–0.769	***<0.001***
Duration from onset to hospitalization (per 1-h increase)	0.987	0.962–1.013	0.328
Hematoma volume (per 1-ml increase)	1.012	1.000–1.025	***0.046***
Hematoma location (supratentorial vs. infratentorial)	0.529	0.221–1.267	0.153
Presence of hematoma expansion (yes vs. no)	1.504	0.756–2.992	0.245
Neutrophil (per 1 × 10^9^/L increase)	1.019	0.940–1.105	0.646
Monocyte (per 1 × 10^9^/L increase)	3.734	1.283–10.869	***0.016***
NLR (per 1-point increase)	1.021	0.987–1.057	0.225
SIRI (per 1-point increase)	1.072	1.020–1.126	***0.006***

*OR, odds ratio; GCS, Glasgow Coma Scale; IVH, intraventricular hematoma; NLR, neutrophil-to-lymphocyte ratio; SIRI, systemic inflammation response index. Significant findings are expressed in bold and italic*.

As shown in Table 4, in the PSM cohort, GCS score (OR, 0.611; 95% CI 0.508–0.736; *p* < 0.001), hematoma volume (OR, 1.034; 95% CI 1.012–1.057; *p* = 0.002), neutrophil count (OR, 1.143; 95% CI 1.030–1.269; *p* = 0.012), NLR (OR, 1.076; 95% CI 1.016–1.139; *p* = 0.012), and SIRI (OR, 1.312; 95% CI 1.096–1.571; *p* = 0.003) were independently associated with 3-month functional outcomes. GCS score (OR, 0.684; 95% CI 0.607–0.770; *p* < 0.001), monocyte count (OR, 35.970; 95% CI, 4.490–288.130; *p* = 0.001), and SIRI (OR, 1.153; 95% CI 1.050–1.265; *p* = 0.003) were independently related to 1-month mortality. NLR was not included in the multivariate logistic analysis because the *p*-value in the univariate analysis did not match the criteria.

**Table 4:** Multivariate logistic regression of included clinical variables for 3-month functional outcome and 1-month mortality in PSM cohort.

**3-month functional outcome**	**OR**	**95% CI**	***P*-value**
GCS (per 1-point increase)	0.611	0.508–0.736	***<0.001***
History of diabetes mellitus (yes vs. no)	2.521	0.272–23.410	0.416
Previous stroke (yes vs. no)	6.588	0.689–62.985	0.102
Hematoma volume (per 1-ml increase)	1.034	1.012–1.057	***0.002***
Hematoma location (supratentorial vs. infratentorial)	1.725	0.682–4.361	0.250
Presence of IVH (yes vs. no)	1.837	0.614–5.499	0.277
Treatment (surgical intervention vs. conservative treatment)	2.326	0.925–5.849	0.073
Neutrophil (per 1 × 10^9^/L increase)	1.143	1.030–1.269	***0.012***
Monocyte (per 1 × 10^9^/L increase)	1.853	0.258–13.333	0.540
NLR (per 1-point increase)	1.076	1.016–1.139	***0.012***
SIRI (per 1-point increase)	1.312	1.096–1.571	***0.003***
**1-month mortality**			
GCS (per 1-point increase)	0.684	0.607–0.770	***<0.001***
Duration from onset to hospitalization (per 1-h increase)	0.990	0.964–1.017	0.469
Presence of hematoma expansion (yes vs. no)	1.766	0.722–4.321	0.213
Neutrophil (per 1 × 10^9^/L increase)	1.002	0.901–1.113	0.977
Monocyte (per 1 × 10^9^/L increase)	35.970	4.490–288.130	***0.001***
SIRI (per 1-point increase)	1.153	1.050–1.265	***0.003***

*OR, odds ratio; GCS, Glasgow Coma Scale; IVH, intraventricular hematoma; NLR, neutrophil-to-lymphocyte ratio; SIRI, systemic inflammation response index. Significant findings are expressed in bold and italic*.

### Predictive Ability of SIRI and NLR in Outcomes

**Figure 2:**
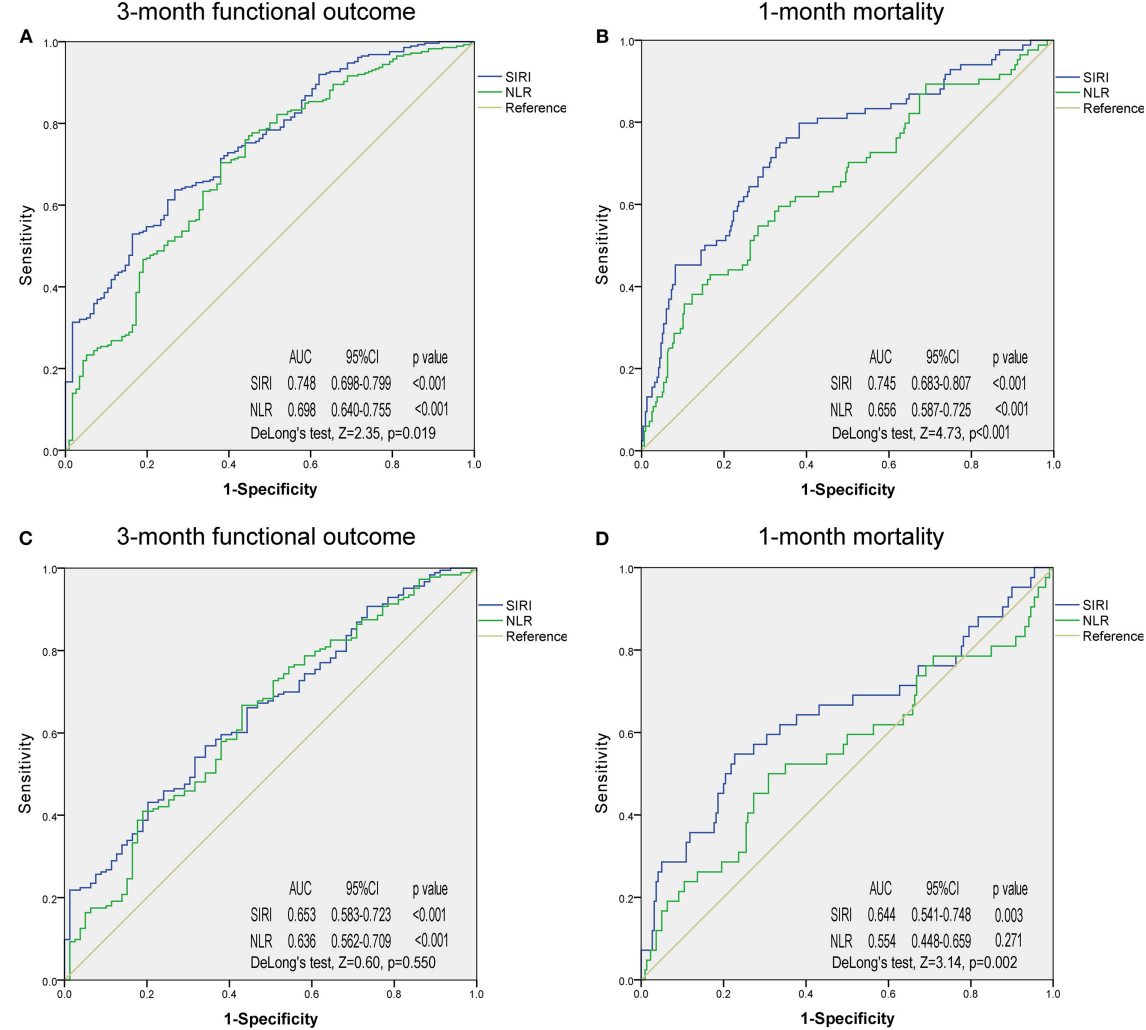
Receiver operating characteristic curves of systemic inflammation response index and neutrophil-to-lymphocyte ratio for predicting 3-month functional outcome and 1-month mortality in the original cohort **(A,B)** and propensity score matching cohort **(C,D)**. SIRI, systemic inflammation response index; NLR, neutrophil-to-lymphocyte ratio; AUC, area under the curve.

ROC analysis was employed to determine and compare the predictive ability of SIRI and NLR in 3-month functional outcomes and 1-month mortality in patients with ICH (Figure 2, Supplementary Figure 1). In the original cohort, SIRI had a stronger predictive ability than NLR in 3-month functional outcome (Figure 2A, AUC 0.748 vs. 0.698; DeLong's test, *Z* = 2.35, *p* = 0.019) and 1-month mortality (Figure 2B, AUC 0.745 vs. 0.656; DeLong's test, *Z* = 4.73, *p* < 0.001). The same applied to the PSM cohort, where the predictive ability of SIRI was also better than that of NLR in 1-month mortality (Figure 2D, AUC 0.644 vs. 0.554; DeLong's test, *Z* = 3.14, *p* = 0.002). In 3-month functional outcome, although predictive ability of SIRI was superior to NLR, there was no statistical difference (Figure 2C, AUC 0.653 vs. 0.636; DeLong's test, *Z* = 0.60, *p* = 0.550).

Predictive models were conducted to further evaluate the predictive accuracy of the aforementioned markers (Table 5). Basic models consisted of independent predictive indicators other than peripheral blood markers. The results indicated that basic models with SIRI had highest C-index and lowest AIC in 3-month functional outcome in both original and PSM cohort, indicating the best predictive accuracy and model-fitting. With regard to 1-month mortality, the basic model with SIRI was superior to that with monocytes in the original cohort, but the result was opposite in PSM cohort.

**Table 5:** Predictive models for predicting primary outcomes of ICH patients.

**Original cohort**	**3-month functional outcome**		**Original cohort**	**1-month mortality**	
**Predictive models**	**C-index**	**AIC**	**Predictive models**	**C-index**	**AIC**
Basic model^§^	0.8551	352.3467	Basic model*^†^*	0.8573	292.8231
Basic model + lymphocyte	0.8568	352.2308	Basic model + monocyte	0.8636	288.2819
Basic model + monocyte	0.8613	347.1597	Basic model + SIRI	0.8668	285.9310
Basic model + NLR	0.8606	349.0812			
Basic model + SIRI	0.8709	335.6420			
**PSM cohort**	**3-month functional outcome**		**PSM cohort**	**1-month mortality**	
**Predictive models**	**C-index**	**AIC**	**Predictive models**	**C-index**	**AIC**
Basic model^‡^	0.8400	244.9730	Basic model^*^	0.8162	181.2230
Basic model + neutrophil	0.8480	239.7489	Basic model + monocyte	0.8421	171.7294
Basic model + NLR	0.8520	239.9733	Basic model + SIRI	0.8339	173.6463
Basic model + SIRI	0.8570	232.8056			

§ *Basic model: GCS, hematoma volume, hematoma location, treatment*.

† *Basic model: GCS, hematoma volume*.

‡ *Basic model: GCS, hematoma volume*.

* *Basic model: GCS*.

*ICH, intracerebral hemorrhage; GCS, Glasgow Coma Scale; C-index, Harrell's concordance index; AIC, Akaike information criterion; NLR, neutrophil-to-lymphocyte ratio; SIRI, systemic inflammation response index*.

## Discussion

In recent years, with the improvement of quality of life and medical conditions, excellent medical treatments, including medication and surgery, have been provided, which have a potent and direct impact on ICH morbidity and mortality (16). Multidisciplinary collaborations, such as between imaging, pathology, physiology, and neurosurgery, are needed to understand this condition and its underlying mechanism. In this study, we focused on the prognostic role of systemic inflammation biomarkers in peripheral blood in patients with spontaneous ICH.

Secondary damage due to ICH in the brain parenchyma induced by inflammatory cells and inflammatory cascades plays a crucial role in disease progression, thus affecting outcomes. Local inflammation adjacent to the primary injury could not be evaluated or measured directly, whereas systemic inflammation might reflect local inflammation in the peripheral blood system to some extent. Damage-associated molecular patterns, which are released by injured or dying neurons and cytokines during early injury, can gain access to the systemic circulation through the broken BBB or cerebrospinal fluid drainage system (22). In animal models of ischemic stroke, immuno-dysregulation after ischemic stroke includes upregulation of systemic inflammatory response. In animal models, a large ICH volume results in decreased leukocytes and lymphocytes and increased monocytes (7). In the same, higher leukocyte counts have been associated to hematoma growth and early neurological deterioration (8). Relevant evidence indicated that the peripheral cellular immune system changed dramatically in the immediate aftermath of ICH (23). Therefore, changes in specific inflammatory markers in the peripheral blood are an indicator of the severity of the primary injury, theoretically. In oncology, inflammatory markers from peripheral blood are used to predict tumor progression and prognosis (24).

We have introduced a novel systemic inflammatory marker SIRI in our study, which was first reported in pancreatic cancer in 2016 (25). Since SIRI and NLR have a great similarity in their components, their predictive abilities in prognosis are compared in this study. NLR has been widely used as an effective indicator and monitor in various diseases, but not limited to tumors, rheumatic diseases, cardiovascular diseases, and infectious diseases (26–29). It is a very sensitive but less specific hematologic parameter to measure stress, intensity of infection/inflammation, and severity of illness of various origin (30). It has also been determined to play a strong predictive role in prognosis for ICH and subarachnoid hemorrhage patients in previous studies (31, 32). Similar to most related studies, the results of this study indicate that NLR is an independent risk factor for 3-month functional outcomes measured by the mRS. Compared with NLR, SIRI is mainly reported in the field of cancer. Recent researches about SIRI in aneurysmal subarachnoid hemorrhage showed that higher level of SIRI served as an independent indicator of unfavorable clinical outcomes (33, 34). In our research, SIRI was superior to NLR in predicting 3-month functional outcomes and has significant advantages in predicting 1-month mortality. However, NLR did not serve as an independent risk factor for 1-month mortality in ICH patients in our study.

Monocytes are mononuclear myeloid cells that originate from the bone marrow and circulate within the bloodstream (35). Like neutrophils, monocyte recruitment in circulation and injured tissues is a key feature of inflammation (36). A previous study has shown that a higher monocyte count on admission is an independent predictor of HE (37). In a study by Walsh et al., absolute monocyte count was independently associated with 30-day case fatality in 240 adult ICH patients, which is consistent with their previous study and our current study (38, 39). In a previous study by Mackey et al., elevated monocyte count was also an independent risk factor for 30-day case fatality (40). In the current study, we also found that monocyte count also served as independent prognostic predictors in 3-month functional outcome and 1-month mortality in the original cohort, and presented an excellent predictive ability in 1-month mortality in the PSM cohort. In consideration of the prognostic ability of monocytes in ICH patients, this could partly explain why the combination of monocyte and NLR gains predictive ability in outcomes.

From another perspective, stability of prognostic capacity in single component including neutrophil, lymphocyte, and monocyte was inferior to SIRI according to the results from multivariate analysis, ROC analyses, and predictive models. In sum, the ability to mirror the extent of inflammation corresponds to the ability to predict prognosis. For peripheral blood-relevant inflammatory markers, diversity compound modes are worth trying and easily realized, which might improve the predictive ability in specific diseases.

In fact, inflammation was not only a prognostic indicator for ICH patients but also a crucial therapeutic target based on the theory that cellular and molecular components of inflammation are involved in post-hemorrhagic secondary brain injury (41). Although the progression of developing specific therapeutic targets remains challenging, markers such as NOD-like receptor family, pyrin domain-containing 3 (NLRP3), C–C chemokine receptor type 1 (CCR1), and Toll-like receptor 4 (TLR4) are proven effective in intervening the progression of ICH-related inflammation (42–44).

There are several limitations to this study. First, follow-up blood tests at each follow-up time point were absent in this study due to incomplete baseline clinical data. For various reasons, it was inconvenient and difficult for some patients to have blood tests regularly, especially when they were not in the hospital. Second, the sample size was not large enough to be divided into training and validation cohorts for further verification. Third, more complicated and comprehensive prognostic patterns are needed to evaluate the prognosis of ICH patients in various aspects, including cognitive function and quality of life. Fourth, not all patients were admitted to hospital within 24 h after onset. Although these patients were in the minority, this might induce unknown bias in laboratory results. Fifth, some occult infections cannot be diagnosed at an early stage by using clinical and laboratory criteria; this might also create bias. Finally, this analysis was conducted in a single institution; therefore, the results should be verified using multi-center data.

## Conclusion

To our knowledge, this is the first study focusing on the prognostic significance of admission SIRI in patients with spontaneous ICH. In this study, SIRI was determined to be an independent predictive indicator for ICH patients in both 3-month functional outcomes and 1-month mortality. Furthermore, its prognostic predictive ability is better than that of NLR. In the near future, multi-center collabora tion is needed to further verify the results and illuminate the underlying mechanism.

## Data Availability Statement

The datasets for this study are available from the corresponding author on reasonable request.

## Ethics Statement

The studies involving human participants were reviewed and approved by Sichuan University. The patients/participants provided their written informed consent to participate in this study.

## Author Contributions

JZ and JL: study design. JL and YY: data acquisition and writing—original draft. JL, XL, and JZ: statistical analysis. JL and ZY: result interpretation. JZ and HL: writing—review and editing. JZ: funding acquisition. All authors contributed to the article and approved the submitted version.

## Funding

This work was supported by the National Natural Science Foundation of China (grant number 81801186), the Science and Technology Department of Sichuan Province (grant number 2020YFQ0009), and the Outstanding Subject Development 135 Project of West China Hospital, Sichuan University (grant number ZY2016102).

## Conflict of Interest

The authors declare that the research was conducted in the absence of any commercial or financial relationships that could be construed as a potential conflict of interest.

## Publisher's Note

All claims expressed in this article are solely those of the authors and do not necessarily represent those of their affiliated organizations, or those of the publisher, the editors and the reviewers. Any product that may be evaluated in this article, or claim that may be made by its manufacturer, is not guaranteed or endorsed by the publisher.

## Supplementary Material

Supplementary Figure 1 Receiver operating characteristic curves of inflammatory markers for predicting 3-month functional outcome and 1-month mortality in the original cohort **(A,B)** and propensity score matching cohort **(C,D)**. SIRI, systemic inflammation response index; NLR, neutrophil-to-lymphocyte ratio; AUC, area under the curve.

## Abbreviations

ICH: intracerebral hemorrhage
BBB: blood–brain barrier
NLR: neutrophil-to-lymphocyte ratio
SIRI: systemic inflammation response index
GCS: Glasgow Coma Scale
mRS: modified Rankin Scale
HE: hematoma expansion
ROC: receiver operating characteristic
PSM: propensity score matching
OR: odds ratio
AUC: area under the curve.
