# Supplementary figures and images for: Prognostic Significance of Admission Systemic Inflammation Response Index in Patients With Spontaneous Intracerebral Hemorrhage: A Propensity Score Matching Analysis
Source: Front Neurol. 2021 Sep 24;12:718032. doi: 10.3389/fneur.2021.718032 (PMC8497988; doi:10.3389/fneur.2021.718032)

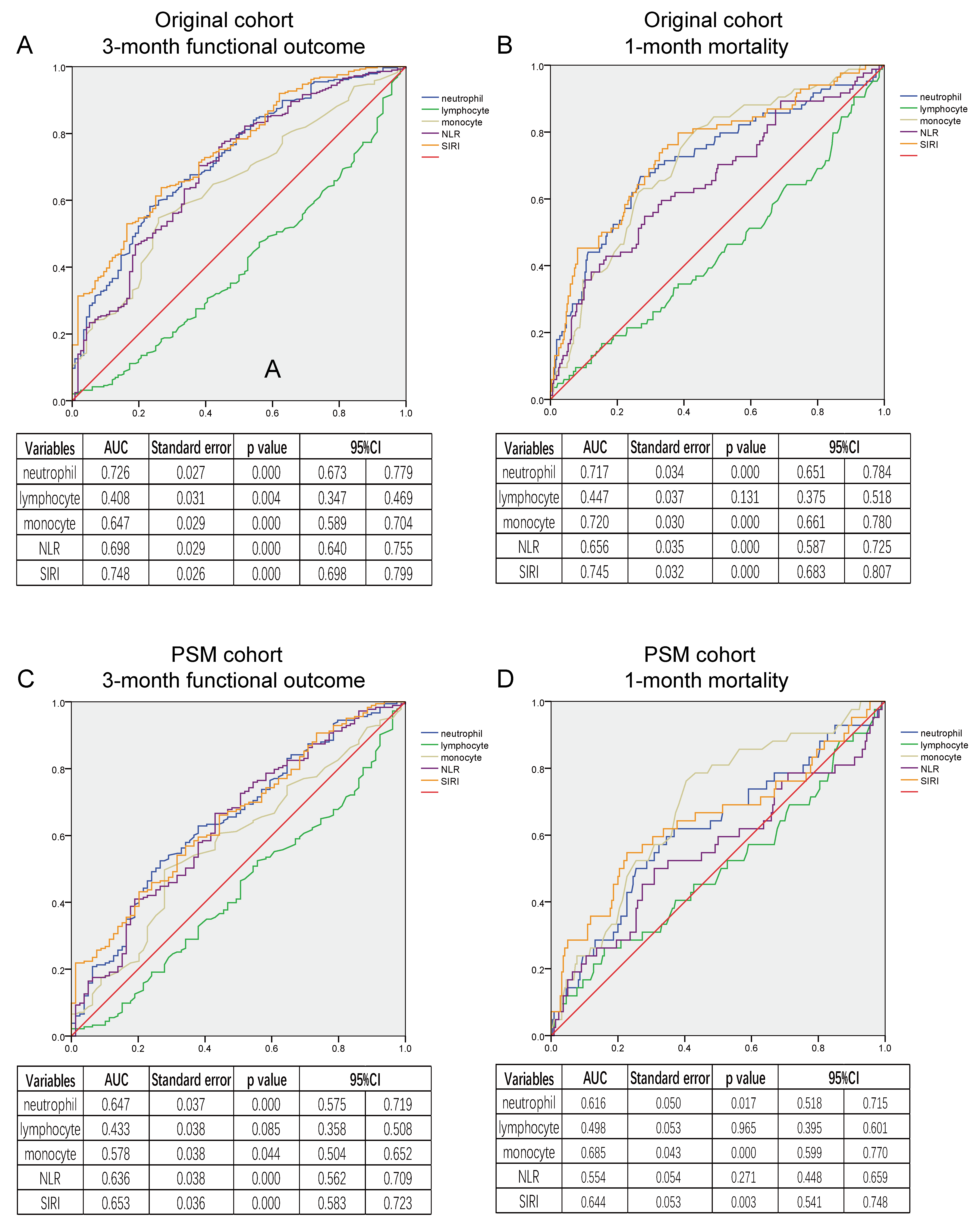

Supplement: Supplementary Figure 1 — Receiver operating characteristic curves of inflammatory markers for predicting 3-month functional outcome and 1-month mortality in the original cohort (A,B) and propensity score matching cohort (C,D). SIRI, systemic inflammation response index; NLR, neutrophil-to-lymphocyte ratio; AUC, area under the curve. [file Image_1.TIF]
